# Defects in the Lunacy Law: Some Changes Held to Be Desirable

**Published:** 1918-05-11

**Authors:** 


### DEFECTS IN THE LUNACY LAW.

### Some Changes held to be Desirable.

The Medico-Psychological Association has appointed a committee "to consider the best method of securing the legal changes which have been long overdue," and presumably of deciding what changes are desirable. There seems to be no immediate prospect of legislation on this or on any other matter, but no doubt it is well to be prepared beforehand, and it may lighten the labours of the committee if we indicate some of the changes in the law that seem to us desirable.

Persons who suffer from mental disease which does not amount to insanity are permitted by the Lunacy Act, 1890, to reside as voluntary boarders in licensed houses, and since that Act was passed very many persons have availed themselves of this privilege, and by the care and treatment they have thus received an impending attack of insanity has in many cases been averted.

The permission given by the existing law extends, however, to registered hospitals and licensed houses only. Persons who are reluctant to mingle with the insane, and persons who have not the means to reside in a registered hospital or licensed house are excluded from the benefit of these provisions of the law.

Experience shows that there are many persons who suffer from mental disease not amounting to insanity who would gladly place themselves under treatment if they could place themselves in private care, but who shrink from entering an institution for the insane. Persons who are competent to treat such cases in private houses can only do so by risking a prosecution for receiving for payment an "alleged lunatic." The term "alleged lunatic" has never been legally defined, and the judgment of medical practitioners as to what does and what does not constitute certifiable insanity is widely discrepant. It very often happens, therefore, that a person who receives into his house for treatment a patient suffering from mental disease, may on the one hand be breaking the law without knowing that he does so, or on the other may, from timidity, refuse to receive a patient who would benefit from his care, and whose reception would not be illegal. One object of any new law should be to regularise these cases and remove these doubts.

Other persons who suffer from mental disease not amounting to insanity and whose mental disease might, by early treatment, be prevented from worsening, have not the means to enter as voluntary boarders into registered hospitals or licensed houses. In many cases such persons have been, in a previous and more severe attack, certified lunatics in public asylums. They have no horror of asylum life, and would willingly place themselves under the protection of a public asylum if the law allowed; but it does not. They cannot be certified as insane, and the law admits no boarders into public asylums. Their lot is a hard one, for neither hospitals for the sick nor public asylums will receive them, and their only course is to wait, getting worse, until they are fit to be certified as lunatics. Another object of any new law should be to meet these cases.

There is a considerable number of persons who can be certified as insane, but whose insanity, if treated promptly, is of short duration; many of these persons are young, and their parents or friends do not recognise that they are insane; or if they do recognise the insanity, they are extremely reluctant to "ruin," as they suppose, the lives of the patients by having them certified as insane. The efficient treatment of such cases is therefore postponed, to the great detriment of the patient, and the serious diminution of his prospect of recovery. There is good reason to suppose that if such persons could be legally placed under care for a short time without the necessity of fully certifying them as lunatics, their illness would be abbreviated, and many cases that are now sent to asylums would be restored to health without that necessity. A third object of any new law should be to legalise temporary treatment of recent cases of insanity without full certification.

There is further a considerable number of persons who are in a sense certifiable as lunatics under the Lunacy Act, 1890, but who are not certified because, from the absence of delusions, or from the inexperience of the medical practitioner, he does not recognise the certifiability, and refuses to certify. These persons remain at large, and the Commissioners have no knowledge of them. The family doctor, who is unable to certify them as insane, would have no hesitation in certifying that their sanity is doubtful, and by becoming voluntary boarders they would be brought to the notice of the Commissioners in Lunacy, who could require them to be certified. A fourth object of any new law should be to secure this result.

Voluntary boarders may, under the Lunacy Act, 1890, be received into licensed houses, but only after the consent of two Commissioners or of two visiting justices has been obtained. This consent is not given by the Commissioners until after a report from the manager of the licensed house that the patient is fit to be received as a voluntary boarder, nor unless the would-be boarder himself writes to them for their permission. Patients fit to be received as voluntary boarders have a natural reluctance to ask for this consent, for many of them regard the request as an admission that they are lunatics; and if the object of the enactment is to secure that the intending voluntary boarder is not a lunatic, and is fully able to appreciate the step he is taking, this object could be equally well secured by requiring the certificate of a doctor that the patient is competent. No such consent is required for the admission of voluntary boarders into registered hospitals, and the omission is not found in practice to have any disadvantage, but quite the reverse.

**DEFECTS IN THE LUNACY LAW—(*continued*).**

There is in the existing lunacy law another provision that is not only pernicious, but absurd, and should be swept away. This is the enactment that the medical certifier shall conduct his examination of the patient "separately from any other practitioner." When the patient is, as sometimes happens, himself a medical practitioner, it is difficult to comply with this regulation, and in almost every case it is embarrassing and absurd. The purpose of the enactment is presumably to prevent collusion and conspiracy by the certifiers to certify the patient wrongfully, and to send a sane man to an asylum. In fact, it has never been even alleged that during the seventy-three years the Lunacy Acts have been in force any patient has ever been wrongfully sent to an asylum by the collusion or conspiracy of two practitioners; and supposing two practitioners were minded to conspire, is it to be supposed that it would be impossible to conspire except in the presence of the patient? Is that the place they would be likely to select for the purpose? Is it not the place that of all others would be avoided? The patient, in most such cases, pays for a consultation, and he is entitled to get what he pays for; and no consultation is of much value, especially in a case of insanity or alleged insanity, unless it is based upon a joint examination of the patient by both or all of the consulting practitioners. Rightly understood, a joint examination is the most secure safeguard that could be devised against improper, hasty, or mistaken certification. And in practice this provision of the law is never complied with. It is always complied with in the letter, inasmuch as the certifying practitioners take care to spend each of them a short time alone with the patient. It is never complied with in the spirit, for their certificates are never based wholly upon this separate examination. The separate examination is an empty form; it may be said without exaggeration to be a farce. If it were complied with in spirit, it would be disadvantageous to the patient, both medically and legally. The provision should be abolished.

